# Functional Quality Characteristics of the Meat from a Dual-Purpose Poultry Crossbreed Suitable for Backyard Rearing in Comparison to Commercial Broilers

**DOI:** 10.3390/foods12132434

**Published:** 2023-06-21

**Authors:** Rekha Sharma, Renuka Sehrawat, Sonika Ahlawat, Vivek Sharma, Mohan Singh Thakur, A. K. Mishra, Reena Arora, M. S. Tantia

**Affiliations:** 1 ICAR-National Bureau of Animal Genetic Resources, Karnal 132 001, India; renuka.sehrawat@gmail.com (R.S.); sonika.ahlawat@icar.gov.in (S.A.); anil.mishra2@icar.gov.in (A.K.M.); reena.arora@icar.gov.in (R.A.); madhu.tantia@icar.gov.in (M.S.T.); 2 ICAR-National Dairy Research Institute, Karnal 132 001, India; vivek.sharma@icar.gov.in; 3 Department of Animal Genetics and Breeding, Nanaji Deshmukh Veterinary Science University, Jabalpur 482 001, India; drmohansingh@gmail.com

**Keywords:** antiglycation potential, antioxidant capacity, carnosine, chicken breast and thigh meat, Indian poultry

## Abstract

Backyard poultry farming contributes to food security, nutrition, and the regular income of rural farmers in India. Their products have a niche market here and fetch higher prices than those of commercial poultry. Improved varieties are being developed to overcome the slow growth, late sexual maturity, and low production of indigenous breeds, while retaining their positive attributes. A comprehensive study was conducted to analyze the functional attributes of meat from the Jabalpur color (JBC), a colored, improved dual-purpose synthetic line, developed by Nanaji Deshmukh Veterinary Science University, Jabalpur, India. The birds were managed in a deep litter system under a backyard type of housing (night shelter and free range). Primal meat cuts (breast and thigh) of the male birds (*n* = 20/group) were evaluated at the age of marketing. The corresponding attributes were compared with the results obtained for commercial Cobb (400) broilers. The protein concentration of JBC breast (25.65 ± 0.39 g/100 g of tissue) and thigh (19.04 ± 0.23 g/100 g of tissue) meat was superior (*p* ≤ 0.05) to that of Cobb broilers. Established assays (in vitro) identified a better (*p* ≤ 0.05) antioxidation capacity in the JBC meat. High-performance liquid chromatography confirmed a considerable quantity of functional biomolecules (carnosine, anserine, and creatine) in the JBC breast and thigh meat extracts. The average carnosine concentration (mg/g of tissue) was 2.66 ± 0.09 and 1.11 ± 0.04 in the JBC breast and thigh meat, respectively. The mRNA expression was quantified by qRT-PCR for the carnosine-related genes: β-alanine transporter (*SLC36A1*), carnosine-synthesizing enzyme (*CARNS1*), and carnosine-degrading enzyme (*CNDP2*); this explained the comparable carnosine in the JBC and Cobb meat. Meat extracts from both genetic groups (JBC and Cobb) had high anti-glycation potential. Higher protein content and antioxidant capacity, along with the bioactive dipeptides in the JBC meat, herald exciting research opportunities for its use in improving the traditional backyard poultry farming system.

## 1. Introduction

Chicken is the most consumed meat across the globe [1]. It is considered a healthy food due to its low energy value, reduced fat content, and higher proportion of polyunsaturated fatty acids [2]. Consequently, the commercial chicken production sector, which is largely based on the indoor rearing of fast-growing hybrid broiler chickens, has grown by leaps and bounds. A new trend in chicken meat consumption is in vogue, where consumers are demanding meat originating from a production system that ensures animal welfare, environmental responsibility, better sensory quality, and health benefits [3,4]. Therefore, free-range chicken production based on slow-growing strains is emerging as the upcoming segment of the contemporary poultry sector. The backyard poultry production system (BPPS) traditionally involves indigenous breeds adapted to free-range backyard conditions and is practiced by 80% of the world’s rural population [4]. The birds have negligible health care requirements, very high broodiness efficiency, and the ability to adapt to adverse environments as well as to defend themselves against predators [5]. However, the mortality rates of the chicks are high due to diseases, predation, malnutrition, and climate adversity [6]. Their meat has the preferred sensory attributes as it is darker, firmer, and more strongly flavored than the meat of commercial broilers [7]. The expectation of health benefits fetches a better price for the meat [8]. Hence, the BPPS plays a pivotal role in the food, nutritional, and financial security of rural people.

Countries, mainly low- and middle-income ones, are making sincere efforts to develop improved backyard poultry to overcome the slow growth, late sexual maturity, and poor production of indigenous breeds [9]. An improved poultry germplasm which is suitable for the BPPS has been developed either through the selective breeding of indigenous birds or through crossbreeding [10]. Poultry crosses gain from the growth rate and feed conversion efficiency of broilers, while disease resistance, colorful plumage, adaptability to harsh climatic conditions, and meat quality attributes are conferred by the native birds [9,11]. Commercial success based on slow-growing chicken breeds has been reported in a number of countries, including France, the UK, the Netherlands, and Germany [1]. The backyard poultry population in India is 317 million, which represents an increase of 45% in the last few decades [12]. Thus, the country can gain by exploiting the new alternative chicken meat and meat products market. However, there is a need to introduce an improved germplasm into the BPPS [13]. Accordingly, an improved dual-purpose synthetic line, Jabalpur color (JBC), was developed under the All India Coordinated Research Project of the Indian Council of Agricultural Research by crossing the broiler control line with the dwarf male line. It was maintained by intersemating at Nanaji Deshmukh Veterinary Science University, Jabalpur, Madhya Pradesh, India. The gain in body weight and egg production of the JBC was better than that of the indigenous backyard poultry breeds. Furthermore, these can be utilized as a parental line to produce commercial color chicks that are suitable for rural and tribal areas [14].

Meat is getting immense scientific attention these days due to the functional benefits of its consumption. Beneficial constituents of enormous physiological significance include proteins, functional amino acids (e.g., taurine and hydroxyproline), histidyl dipeptides (e.g., carnosine and anserine), and creatine (a metabolite of amino acids). Carnosine and anserine are potent antioxidants [15], whereas creatine is both an antioxidant and a major component of energy metabolism in the brain and skeletal muscle [16]. The abundance of histidyl dipeptides contributes to the superior antioxidant capacity of chicken meat [17], which is best compared to pork, beef, and fish [18]. Scientific evidence is mounting regarding the critical roles of these bioactive molecules in preventing oxidative stress and damage to mammalian cells [19,20,21,22]. Meat quality is a complex trait that is influenced by genetic and environmental factors [23]. Differences in nutritional, carcass, and meat quality traits among slow-growing and fast-growing chicken lines have been reported in the literature [12]. The meat of slow-growing indigenous chickens had a higher protein content, a better amino acid profile, and lower fat and cholesterol and was rich in functional biomolecules such as carnosine [24,25]. Variation in the histidyl dipeptide content of the breast and thigh meat has also been reported [25]. To date, there are limited data on the functional attributes of indigenous backyard poultry meat [24,26], while hardly any information is available for the chicken lines.

Based on the aforementioned premises, the present study compared the antioxidant capacity, antiglycation potential, concentration of bioactive antioxidants (carnosine, anserine, and creatine), and expression of genes related to carnosine accumulation in the breast and thigh meat of Jabalpur color (JBC) to those of a commercial Cobb broiler.

## 2. Materials and Methods

### 2.1. Ethics Statement

In vivo experiments were not conducted in the present study. The ethics committee of the Veterinary College, Nanaji DeshmukhVeterinary ScienceUniversity, Jabalpur, approved the experimental design and permitted the study (Order number 4040/Dean/Vety/2018, date 18 December 2018). The meat samples were collected as per the guidelines and regulations of the ethics committee.

### 2.2. Birds and Meat Sample Collection

**Figure 1:**
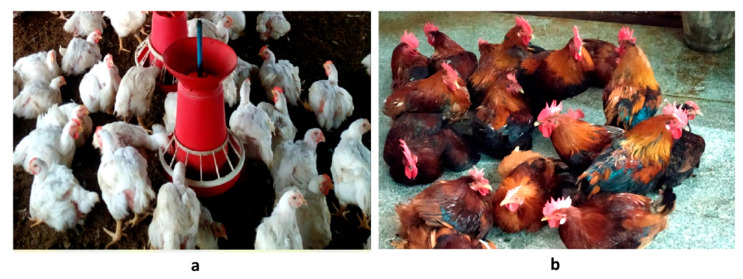
Representative flocks and birds of the Cobb 400 broiler (**a**) and Jabalpur color (**b**).

The male birds of the Jabalpur color (JBC) chicken and the commercial Cobb 400 broiler (Figure 1) (N = 20, each) were maintained at the poultry farm of the College of Veterinary Sciences and Animal Husbandry, Jabalpur (23°10′1.09″ N 79°57′0.22″ E), India. The birds were reared under identical management conditions in the open-sided poultry house. The birds had free access to water and were fed ad libitum with the standard formulated diets prescribed by the Indian Council of Agricultural Research [27]. The ingredients in the diets of the Cobb and JBC were the same, with soy meal as the main source of protein. The Cobb broiler birds were fed a diet formulated with 21.5% crude protein (CP) and 3000 kcal/kg of metabolizable energy (ME) up to 5 weeks of age; subsequently, they were fed a diet formulated with 19.5% CP and 3000 kcal/kg of ME until 8 weeks of age. Similarly, the JBC birds were fed a diet with 19% CP and 2700 kcal/kg of ME until 8 weeks of age and 16.5% CP and 2600 kcal/kg of ME until 20 weeks of age.

Standard scientific procedures were followed to sacrifice the birds at the age of marketing (20 weeks for the JBC and 8 weeks for the Cobb), following the guidelines of the ethics committee. The breast (pectoralis major muscle) and thigh (biceps femoris muscle) meats were dissected. Both of the primal cuts were trimmed of visible fat and connective tissue. The cleaned meat sample was divided into two portions. One half was used for biochemical analysis and the other was used for the expression proofing of genes. The sample to be used for biochemical analysis was wrapped in an aluminum sheet to avoid exposure to light, packed in PE plastic bags, and frozen at −80 °C until further analysis. The portion to be used for RNA isolation was finely chopped and added to the cryovials filled with RNALater^®^ (Ambion Inc., Austin, TX, USA). The cryovials were kept overnight at 4 °C, followed by storage at −80 °C until further processing.

### 2.3. Analytical Methods

All chemicals were purchased from Sigma-Aldrich (St. Louis MO, USA) unless otherwise stated.

#### 2.3.1. Preparation of Hydrolysate

The thawing of the breast and thigh samples was conducted in a thermostatic bath (20 ± 2 °C). For the preparation of the meat (breast and thigh) extracts of the JBC (*n =* 20) and Cobb (*n =* 20), 2 g of meat was homogenized in 20 mL of phosphate-buffered saline (PBS, pH 7.4) with the help of a homogenizer (Benchmark Scientific D1000, Sayreville, NJ, USA). The samples were kept in the ice bath during the homogenization. The homogenate was extracted in the dark at 4 °C for 20 min followed by centrifugation at 10,000× *g* for 15 min at 4 °C. The supernatant was collected, and the aliquots were stored at −20 °C until further use.

#### 2.3.2. Estimation of Protein, Antioxidant Activity, and Antiglycation Capacity

The Lowry method [28] was used to estimate the protein content using Bovine serum albumin (BSA) as a standard. Absorbance at 660 nm was recorded spectrophotometrically (UV-Vis 2080 Plus; Analytical Technologies Ltd. India). The DPPH (1,1-diphenyl-2-picrylhydrazyl)assay was performed with 1 mL of extract, which was diluted with 1 mL of water and 1 mL of ethanolic DPPH solution (0.2 mM). The mixture was incubated in the dark for 40 min at room temperature. A control with ethanol instead of a sample was also processed simultaneously. The absorbance of the solution was measured at 517 nm after a 10 min centrifugation (4500 rpm) at 4 °C. Ascorbic acid was used as a positive control. The scavenging of DPPH radicals (%) was expressed as

[(Control absorbance-Sample absorbance)/Control absorbance] × 100

The ABTS (2,2-azinobis (3-ethyl-benzothiazoline-6-sulfonic acid) assay for antioxidant capacity was performed as per Re et al. [29], with some modifications. The ABTS^+^ radical was generated by mixing equal volumes of ABTS^+^ (14 mM) and potassium persulfate (5.9 mM) solutions, which were allowed to react for 12 h in the dark at 23 °C ± 1 °C. This was diluted with distilled water (30 °C) to an absorbance of 0.70 ± 0.02 at 735 nm and its bleaching rate by the sample was monitored at 735 nm. Trolox, the hydrophilic homolog of vitamin E (0–600 µM) was used as the size standard, and the inhibition caused by the sample was expressed as the Trolox equivalent antioxidant capacity (TEAC).

The ferric reducing antioxidant power (FRAP) assay measured the ability of the sample to reduce ferric iron to ferrous iron. It was performed using the EIAFECL2 kit (Thermo Fischer Scientific). The FRAP value was derived using the FeCl_2_ standard curve (200–1000 μM), and the results were expressed in mM Fe^2+^/g of meat. The method of Dinis et al. [30] was modified slightly to measure the metal (iron) chelation activity. The formation of Fe^2+^-ferrozine complex was measured at 562 nm in the mixture of extract (100 µL), 2 mM ferrous chloride solution (100 µL), and 200 µL ferrozine (5 mM) after 10 min of incubation at room temperature. the iron chelating activity (% inhibition) was calculated as

[(Absorbance of the control − Absorbance of the sample)/Absorbance of the control] × 100.

A calibration curve (25–200 µM) of ethylene diamine tetra acetic acid (EDTA) was used to express the results as an EDTA equivalent chelating capacity (μM EDTA/g of tissue). The MAK-187 kit (Sigma-Aldrich, Burlington, MA, USA) was selected for the estimation of the ability of a sample to reduce Cu^2+^ ion to Cu^+^, which is also referred to as a cupric reducing antioxidative capacity (CUPRAC) assay. To 40 µL of the sample, 100 µL of Cu^2+^ working solution was added, and the mixture was incubated at room temperature for 90 min in the dark. Absorbance was recorded at 570 nm and the total antioxidant capacity of the meat extract (Trolox equivalents (TE)/g of tissue) was calculated from the standard curve of Trolox.

Similarly, the oxygen radical absorbance capacity (ORAC) assay was performed with the ab233473 ORAC assay kit (Abcam, Cambridge, UK) following the manufacturer’s instructions. In brief, the meat extract samples were dispensed into a 96-well microplate with fluorescein solution (150 μL, 1×) in each well. After thorough mixing, the plate was incubated at 37 °C for 30 min. The microplate was immediately placed in the microplate reader (Model: Infinite F200 Pro, Tecan Austria GmbH, Austria) after the addition of 25 μL of the free radical initiator solution. The decay in fluorescence per minute was recorded for 60 min with an excitation wavelength of 300 nm and emission at 380 nm. A blank that had PBS instead of the extract and an antioxidant standard (Trolox (6-hydroxy-2,5,7,8–tetramethylchroman-2-carboxylic acid) (2.5–50 µM) were processed simultaneously. Each sample was processed in triplicate. The area under the curve (AUC) for each sample and standard was calculated using the final assay values and the linear regression:

$$\frac{AUC}{RFU0}=1+\frac{RFU1}{RFU0}+\frac{RFU2}{RFU0}+\frac{RFU3}{RFU0}+.\ldots \ldots .+\frac{RFU59}{RFU0}+\frac{RFU60}{RFU0}$$

where *RFU*0 = relative fluorescence value of time point zero, and *RFUx* = relative fluorescence value of time (minutes) points. The net AUC was obtained by subtracting the AUC of the blank from the AUC of each sample and standard. The Trolox standard curve was prepared by plotting the net AUC on the Y-axis against the concentration on the X-axis. The regression equation between the net AUC and the antioxidant concentration was calculated. The slope of the equation was used to calculate the µM Trolox equivalents (TE) of the unknown sample (ORAC value) expressed as µM TE/g tissue, the Trolox equivalent antioxidant capacity (TEAC).

The superoxide dismutase enzyme activity was determined using the SOD Assay Kit- 19160 (Sigma-Aldrich, Burlington, MA, USA). The water-soluble tetrazolium salt, WST-1 [2-(4-Iodophenyl)-3-(4-nitrophenyl)-5-(2,4-disulfophenyl)-2Htetrazolium, monosodium salt], inhibition curve was used to calculate the inhibition activity of the SOD by measuring the decrease in the color development at 440 nm.

The method of Abdelkader et al. [31], with slight modifications [24], was followed to compare the in vitroantiglycation capacity of the JBC and Cobb meat extracts. Sodium azide (0.02%) was utilized as the antimicrobial agent. Carnosine (10 mM) and aminoguanidine (30 mM) were selected as the standard glycating agents, while the phosphate buffer was used as a blank. The samples were run in triplicate, and the inhibition (%) of the advanced glycation end products (AGEs) formation by the meat extracts was calculated as follows:

[1 − (FI of extract/FI of control)] × 100, where FI refers to fluorescence intensity).

#### 2.3.3. Estimation of Carnosine, Anserine, and Creatine

Carnosine, anserine, and creatine were quantified in the breast and thigh meat following the method of Mora et al. [32], with minor modifications. The meat sample (0.5 g) was homogenized (3000× *g*) with 0.1 N HCl (3 mL) for 1 min. The homogenate was centrifuged (10,000× *g*) for 20 min at 4 °C. The supernatant was collected and filtered using Whatman No. 4 filter paper. This filtrate (250 µL) was deproteinized with acetonitrile (750 µL) for 20 min at 4 °C. Subsequently, the mixture was centrifuged (10,000× *g*) for 10 min at 4 °C. The supernatant was filtered through a 0.22 µm membrane filter (Millipore, Sigma, St. Louis, MO, USA), and the filtrate was used as the sample for high-performance liquid chromatography (HPLC).

Twenty microliters of the sample was injected into an HPLC system (1260 Infinity; Agilent Technologies, Santa Clara, CA, USA) fitted with the Zic-HILIC silica column (4.6 × 150 mm, 3 μm; Waters, Milford, MA, USA). The temperature of this column was maintained at 35 °C. Two solvents, solvent A (pH 7, 0.65 mM ammonium acetate in water:acetonitrile, 25:75, *v*/*v*) and solvent B (pH 6.8, 4.55 mM ammonium acetate in water: acetonitrile, 70: 30, *v*/*v*), were used as mobile phases. The flow rate was 1.2 mL∙min^−1^ for 8 min with a linear gradient (0% to 100%) from solvent A to solvent B. Carnosine, anserine, and creatine were detected at 214 nm by the diode array detector. Standard curves for the three biomolecules were drawn with their respective standards (Sigma-Aldrich, St. Louis, MO, USA), and the area under the curve (AUC) of the peaks was used to obtain their regression equations. The carnosine, anserine, and creatine contents (mg/g of wet tissue weight) were quantified by plotting the AUC of each sample against its standard curve data.

#### 2.3.4. Quantitative Real-Time Polymerase Chain Reaction (qRT-PCR)

Total RNA from the JBC (*n =* 20) and Cobb (*n =* 20) breast and thigh tissues was extracted using TriReagent (Sigma-Aldrich). The RNA was column purified with the Qiagen RNeasy kit (Cat. No. 74004) following the manufacturer’s instructions. The quantity and quality of the RNA were estimated with a Nanodrop ND-1000 spectrophotometer (Thermo, Scientific, Waltham, MA, USA). The samples with A260/A280 and A260/A230 ratios of more than 2.0 were selected for qRT-PCR analysis. Two micrograms of RNA was reverse transcribed to the cDNA using the SuperScript^®^ III First-Strand Synthesis System (Catalog number: 18080051; ThermoFisher Scientific, Waltham, MA, USA) following the manufacturer’s instructions. To estimate the differential expression of genes related to carnosine accumulation [33], SYBR Green I chemistry was used on a real-time PCR system (LightCycler^®^480 Instrument II, Roche Life Science, Mannheim, Germany). The samples were amplified with the primers (Integrated DNA Technologies, Coralville, IA, USA) of the candidate genes CARNS1 (Carnosine synthase 1), CNDP2(Carnosine dipeptidase2), and SLC36A1 (Solute carrier family 36, member 1) following the protocol described by Sharma et al. [24]. β actin was used as the reference gene, and the relative expression of each gene was quantified (fold change) using the 2^−ΔΔCt^ method [34]. The Graphpad Prism 8.0 software package (https://www.graphpad.com/scientific-software/prism/ (accessed on 30 November 2021)) was used to obtain the graphical output of the relative mRNA expression of the genes.

### 2.4. Statistical Analyses

Three replications of each experiment were performed on the breast and thigh tissues of the Cobb and JBC (N = 20 each). The results are presented as means ± standard error (SE). The data were analyzed using the *t* test. The significance level was set at *p* ≤ 0.05 to indicate a significant difference.

## 3. Results

The functional properties of JBC meat were explored and further compared with those of the commercial Cobb broiler. The two primal chicken meat cuts, breasts and thighs, were investigated.

### 3.1. Antioxidant Capacity Assays

One of the important components contributing to the functional properties of meat is its antioxidative capacity. Thus, the general antioxidant property and capacity of the JBC chicken meat were elaborated with seven commonly used in vitro methods recommended for foods. The antioxidation capacity ofthe JBC chicken meat was reflected by all the assays (Table 1 and Table 2). Moreover, the majority of the assays (4) indicated a better antioxidant capacity of the JBC meat than that of the Cobb broiler (Table 1). The average values of the % inhibition and Trolox equivalent antioxidative capacity for the ABTS (2,2′-azinobis-3- ethylbenzothiazoline-6-sulfonic acid) radical scavenging assay; the % inhibition for the DPPH (1,1-diphenyl-2-picrylhydrazyl) radical scavenging assay;the ferric reducing antioxidant power; and the cupric reducing antioxidative capacity were significantly higher in the JBC breast meat. Only one assay (MCA) indicated the superiority of the Cobb’s breast meat over that of the JBC, while an antioxidant ability of similar magnitude was identified by the ORAC and superoxide dismutase assays (Table 1). The Trolox equivalent antioxidant capacity (µM Trolox equivalent (TE)/g of tissue) of the JBC breast meat was 765.82 ± 9.48, while that in the Cobb’s breast meat was 748.56 ± 7.48. The total antioxidant ability of the thigh meat extract is presented in Table 2. Once again, the DPPH radical scavenging assay, the ferric reducing antioxidant power, the oxygen radical absorption capacity, and the superoxide dismutase activity of the JBC meat were noticeably better than those of the Cobb. The antioxidant capacity of the breast and thigh tissue was found to be different within the same genetic group (JBC or Cobb) (Supplementary Table S1). The breast meat was more potent in antioxidant ability, as reflected by the significantly (*p* ≤ 0.05) higher values for ABTS (2,2′-azinobis-3-ethylbenzothiazoline-6-sulfonic acid), DPPH (1,1-diphenyl-2-picrylhydrazyl), thecupric reducing antioxidative capacity (CUPRAC) assay, and superoxide dismutase activity (Supplementary Table S1).

### 3.2. The Concentration of Selected Functional Molecules

The muscular contents of carnosine, anserine, and creatine were established in the breast and thigh meats by HPLC. The absolute concentrations of these metabolites were estimated from the standard curve regression equation of pure carnosine, anserine, and creatine (Supplementary Figure S1). The R^2^ value for the three standards was very high (0.998). A sizeable amount of carnosine, anserine, and creatine was present in the breast and thigh meat extracts (Table 3). The mean carnosine concentration (mg/g of tissue) in the JBS breast (2.66 ± 0.09) and thigh (1.11 ± 0.04) was of similar magnitude in the Cobb broiler (breast, 2.73 ± 0.1 and thigh, 0.98 ± 0.03). Among the two types of meat cuts, the breast meat had significantly (*p* ≤ 0.05) higher concentrations of carnosine, anserine, and creatine (Table 3). Irrespective of the genetic group, the breast meat had higher protein content than the thigh (Table 3). Additionally, the JBC meat was found to be significantly richer in protein content in comparison to the Cobb broiler.

### 3.3. Expression of the Carnosine-Related Genes

**Figure 2:**
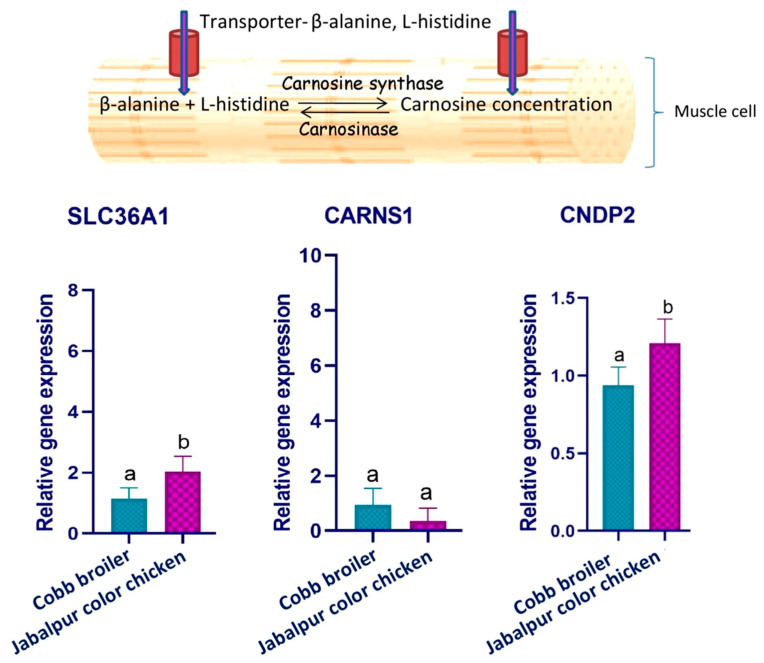
Relative mRNA abundance of SLC36A1, CARNS1, and CNDP2 genes in the breast meat of Cobb broiler and Jabalpur color chicken. Error bars represent the standard deviations from triplicate qRT-PCR runs. Different letters denote significant difference (*p* ≤ 0.05).

The molecular machinery of the carnosine concentration in the muscle was explored with qRT-PCR. The expression of the three genes was quantified. These included a gene coding for the transporter of carnosine building blocks inside the cell (Solute carrier family 36, member 1-SLC36A1); a gene coding for the enzyme catalyzing the synthesis of carnosine from its building blocks (Carnosine synthase1-CARNS1); and a gene coding for the enzyme that degrades muscular carnosine into its building blocks (Carnosine dipeptidase-CNDP2). The comparative gene expression profile of the JBC and Cobb breast meat (Figure 2), revealed that CARNS1 was expressed at a similar magnitude, while SLC36A1 and CNDP2 were differentially expressed between the JBC and Cobb. While the SLC36A1 expression was significantly higher, the CNDP2 expression was significantly lower (*p* ≤ 0.05) in the JBC as compared to the Cobb broiler.

### 3.4. The Antiglycation Potential of Meat Extract

**Figure 3:**
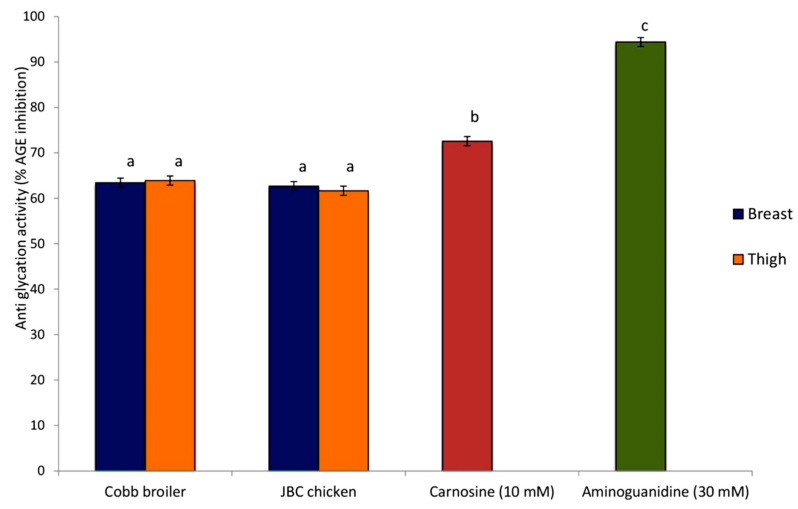
Antiglycation effect of breast and thigh meat extracts in the bovine serum albumin–methylglyoxal model. Aminoguanidine (30 mM) served as the positive control. Bars represent mean ± standard error (*n =* 20) and different letters denote significant differences (*p* ≤ 0.05).

The high efficiencyof the BSA–MGO (bovine serum albumin–methylglyoxal) system selected for measuring the antiglycation potential was confirmed by the 94.39 ± 2.32% AGE inhibition recorded with the positive control (30 mM aminoguanidine). The JBC and Cobb broiler meat extracts also depicted the ability to inhibit the formation of fluorescent AGEs (Figure 3). They had a high antiglycation potential (>60%) since the corresponding value with pure carnosine was 72.57 ± 2.12. Breast and thigh tissue were equally potent in inhibiting the AGEs in both genetic groups (JBC and Cobb) as the percentage inhibition of the AGEs was 62.68 ± 0.86 and 61.66 ± 0.87 for the JBC breast and thigh meat, respectively. Similarly, the Cobb breast and thigh meat inhibited the AGEs by 63.43 ± 0.89 and 63.91 ± 0.98 percent, respectively.

## 4. Discussion

The antioxidant activity of meat or any food is considered to be a valuable index of both the antioxidant status of the animal and its potential health benefits for consumers. Thus, the antioxidative capacity is an important indication of the functional properties of the meat. It can also predict the susceptibility of meat to oxidative degeneration, one of the main causes of spoilage [35].

Antioxidants play a vital role in both the human body and the food system to lessen the harmful effects of free radicals and oxidative processes [36]. Reactive oxygen species (ROS) are either produced during normal cellular metabolism or are the result of stress [37]. An imbalance between ROS and antioxidant defenses culminates in oxidative stress that can harm key biomolecules (proteins, carbohydrates, lipids, and nucleic acids). This contributes to the pathogenesis of several human diseases, including diabetes mellitus, chronic inflammation, atherosclerosis, neurodegenerative disorders, and certain types of cancer [38]. Meat is a rich source of endogenous enzymatic (e.g., superoxide dismutase, catalase, glutathione peroxidase, glutathione reductase) and non-enzymatic antioxidants (L-carnosine, β-tocopherol, L-ascorbic acid, etc.). Hence, antioxidant activity in meat extracts is a cumulative effect of various biologically active compounds that exert their action through a variety of mechanisms (viz., preventing chain initiation by scavenging radicals, inhibiting the propagation of radical chain reactions, decreasing localized oxygen concentration, metal chelation, and decomposing peroxides). Considering these factors, more than one method needs to be employed to verify the antioxidant activity [36]. Therefore, the antioxidant activity of the breast and thigh meat extracts was quantified by the seven in vitro methods (Table 1) that are most commonly used for determining the antioxidant capacity of food in vitro [39]. The general principle of these methods was that a redox-active compound or a synthetic colored radical was generated, and the ability of a meat extract to reduce the redox-active compound or to scavenge the radical was monitored. An appropriate standard was utilized to quantify the antioxidant capacity [40].

The results suggest that JBC poultry meat could significantly contribute to the antioxidant activity of the diet. It would be a better alternative to the commercial broiler in this respect (Table 1). Breast tissue has a much better antioxidant capacity than the thigh (Supplementary Table S1). These conclusions were drawn after explicit experimentation. The antioxidant capacity of food involves hydrogen atom transfer (HAT) or single electron transfer (SET). The SET and HAT mechanisms almost always occur together, with the balance determined by the structures of the antioxidants and the prevailing pH. The conclusions drawn here are based on both the SET-based (TEAC, FRAP, DPPH, ABTS, CUPRAC, MCA) and HAT-based (ORAC) methods. While the SET-based methods detected the ability of the meat extract to transfer one electron to reduce any compound, including metals, carbonyls, and radicals [41,42], the HAT-based methods measured the classical ability of an antioxidant to quench free radicals by hydrogen donation (ORAC). The ABTS methods used both HAT and SET mechanisms [43]. Additionally, the superoxide anion radical scavenging assay measured the sample’s enzyme-based scavenging ability for oxidants, which can interact with and damage the major macromolecules in food items [44]. Overall, multiple approaches increased confidence in the obtained results.

HCDs (carnosine and anserine) are considered to be effective hydrophilic antioxidants. Chickens have previously been reported to have high skeletal muscle HCD content [17,45] due to their primary role as intracellular proton buffers. Functional imidazole rings of dipeptides can readily donate hydrogen to free radicals, which are then converted to non-radical moieties [46]. This capability is further augmented by the β-alanine constituent of dipeptides. The antioxidant action is due to both the radical scavenging activity and the metal chelating properties [35]. Copious and similar amounts of all these functional molecules were present in both the JBC and the Cobb broiler meat (Table 3). Anserine is more prevalent in nature than carnosine, including in chicken meat [47]. The present results were along the same lines (Table 3).

The comparable magnitude of carnosine across the two genetic groups was explained by the expression profiles of the concerned genes. Muscle carnosine concentration is mainly governed by two enzymes. The intracellular ATP-dependent carnosine synthetase catalyzes the synthesis of carnosine from β-alanine and histidine, while carnosine is hydrolyzed by carnosinase-2 [48]. The expression of the carnosine-synthesizing enzyme (CARNS1) gene was similar across the two groups (Figure 2). The expression of the β-alanine transporter gene (SLC36A1) was significantly higher (*p* ≤ 0.05) in the JBC muscle cells, which can facilitate the added availability of β-alanine for the carnosine synthesis. However, significantly higher (*p* ≤ 0.05) expression of the gene coding for the carnosine-degrading enzyme (CNDP2) in the JBC muscle (Figure 2) might have balanced the anabolic effect.

The breast meat was much more enriched in HCDs than the thigh meat (Table 3). The results are in agreement with the published reports of lower HCD content in the thigh meat [17,24]. HCD abundance in the breast meat is attributed to the limited aerobic capacity of the predominantly glycolytic chicken breast muscles, which require the biological capability to protect against the effects of acidosis [49]. HCDs delay muscular fatigue and enhance the ability to catch prey or escape predators. In general, carnosine is more abundant in the white muscles than in the red muscles, and in type II muscle fiber than in type I muscle fiber [50]. Breast meat is categorized as white meat with mainly type II muscle fiber, while thigh meat belongs to the second category [51]. Differential expressions of genes for the key transporter and enzymes (SLC36A1, CARNS1, CNDP2) that are involved in muscle cell carnosine homeostasis support the higher accumulation of carnosine in the breast [24].

Glycation is implicated as the major underlying cause of multiple chronic human complications, viz., diabetes, cardiovascular disease, nephropathy, neuropathy, and retinopathy [52]. Glycation results in the accumulation of a heterogeneous group of biomolecules that are collectively termed advanced glycation endproducts (AGEs). The interaction of the AGEs with the plasma membrane receptors modifies intracellular signaling, which alters gene expression, the accumulation of free radicals, and the release of pro-inflammatory molecules [53]. In our investigation, we found positive influences of both types of meat extracts (breast and thigh) on the inhibition of AGEs (Figure 3). The bovine serum albumin–methylglyoxal (BSA–MGO) system which was selected as MGO is known to be a very reactive precursor of AGE formation and has been extensively used as an in vitro marker for oxidative cleavage product formation [54]. The extracts of meat from the two genetic groups confirmed them as effective glycation inhibitors (Figure 3). The high antiglycation potential of chicken meat is likely to be related to the high concentration of carnosine and its derivatives in their muscles, as carnosine was able to inhibit the formation of fluorescent AGEs by 72.57%. Carnosine has been described as a very good inhibitor of AGE formation and can even reverse previously formed AGEs [55]. Likewise, anserine had a trans-glycating effect similar to that of carnosine [56]. Thus, the superior antiglycation potential can be considered to be one of the functional attributes of chicken meat. Carnosine and anserine also provide an additional benefit by scavenging ROS. Better antioxidant capacity further contributes to meat quality by reducing the oxidative stress-related ill effects. Hence, foods containing these peptides could be used to improve health.

The potential applications of carnosine and related dipeptides are wide-ranging and extend widely into the fields of agriculture, zoology, sports sciences, etc., while the potential therapeutic applications are in neurology, diabetes, cardiovascular disease, nutrition, etc. [17]. The abundance of carnosine in skeletal muscle has stirred the scientific imagination and research over the past century. Extraordinary efforts were made in the last decade once it was established that the dietary intake of carnosine enhances its concentrations in skeletal muscles, the brain, and the heart [57]. The dietary intake of the carnosine precursor beta-alanine also augmented human muscle carnosine concentration and improved exercise capacity [58,59,60]. The oral administration of carnosine enhanced the total antioxidant capacity (TAC) level of serum in humans [61]. The research utilizing both in situ and in vivo techniques suggests that carnosine has good bio-accessibility and that it is readily absorbed from the diet. The researchers found a rapid increase in plasma carnosine after the ingestion of grilled beef top loin or stewed beef that declined back to basal levels in 6 to 7 h post-feeding [62]. Moreover, carnosine and anserine are not degraded completely by food processing operations; thus, they are available for absorption, and they confer health benefits upon consumption [63]. Dietary creatine is also important in maintaining good human health [64]. The synthesis of creatine falls short of its requirement under several physiological (pregnancy, lactation, exercise, etc.) and pathological (diabetes, ischemia, injury, etc.) conditions [65]. Creatine supplementation in humans has an ergogenic effect, and it improves cognitive function and enhances muscle functional capacity [20,66]. Taken together, all three biomolecules are considered naturally occurring dietary functional food components, and chicken meat is a very good source that can be considered as a functional food for human consumption.

Growing awareness is resulting in an increasing demand for food that has health benefits and disease prevention potential. Consequently, antioxidants have become vital in food preservation and present-day health care. Foods that are rich in natural antioxidants are in vogue these days due to doubts over the toxic effects of synthetic antioxidants [36]. JBC meat is a good source of dietary protein, carnosine, anserine, and creatine. These functional biomolecules play a vital role in inhibiting oxidative stress and, hence, chronic diseases, inflammation, and tissue injury and in improving metabolic profiles in animals and humans. The in vitro anti-oxidation and anti-glycation potential of JBC meat was identified in the present study. The results on the functional attributes will help in generating an optimistic perception of the improved backyard poultry germplasm among the niche market consumers who prefer traditional backyard poultry meat for its superior human health-related properties. Such an informative update on JBC meat will educate and positively influence the public and policymakers about the improved backyard poultry germplasm.

## 5. Conclusions

Thedual-purpose improved backyard chicken (Jabalpur color) meat had better nutritional and functional quality in comparison to the meat from commercial broilers. It had significantly higher protein content and a better antioxidant capacity. The results of this study are meaningful to breeders, producers, and consumers. These results can be exploited for the marketing of meat from improved backyard poultry varieties that are developed to bridge the gap between the quality and quantity of meat obtained from indigenous chickens and commercial broilers, respectively.

## Figures and Tables

**Table 1:** In vitro antioxidant capacity assays in breast meat of Cobb broiler and Jabalpur color chicken (*n =* 20/group).

Sr No	Antioxidant Capacity Assays	Unit of Measurement	Genetic Group								*p*-Value
			Cobb Broiler				Jabalpur Color Chicken				
			Mean	SE	Minimum	Maximum	Mean	SE	Minimum	Maximum	
1	ABTS (2,2′-azinobis-3- ethylbenzothiazoline-6-sulfonic acid) radical scavenging assay	% Inhibition	43.78	1.47	33.33	57.58	52.12	1.36	43.94	63.64	*
	TEABTS (Trolox equivalent antioxidative capacity)	Trolox equivalent (TE) µM/g of tissue	6062.5	257.31	3350	8350	7375	210.65	6100	9350	*
2	DPPH (1,1-diphenyl-2-picrylhydrazyl) radical scavenging assay	% Inhibition	70.06	0.59	60.51	74.30	73.92	0.44	68.80	76.63	*
3	FRAP (ferric reducing antioxidant power)	mM Fe^2+^/g tissue	15.24	0.40	11.6	18.5	22.84	0.25	20.7	24.1	*
4	Cupric reducing antioxidative capacity (CUPRAC) assay	Trolox equivalent (TE) mM/g of tissue	9	0.24	7.219	10.438	12.71	0.32	10.75	16.32	*
5	ORAC (oxygen radical absorption capacity) assay	Trolox equivalent (TE) µM/g of tissue	748.56	7.48	713.02	776.0	765.82	9.48	730.02	803.07	NS
6	MCA (metal chelation activity)	% Inhibition	53.63	1.79	40.22	65.71	46.3	2.36	30.5	63.41	*
		EDTA equivalent activity— EEA µM/g of tissue	2819.29	85.84	2170.02	3400.03	2468.22	112.81	1712.03	3290.30	*
		Carnosine equivalent activity—CEA mM/g of tissue	148.63	4.34	116.03	178.24	130.89	5.70	92.75	172.62	*
7	Superoxide dismutase activity	% Inhibition	93.16	1.07	83.25	99.62	95.84	0.93	88.12	99.87	NS

SE: standard error of the mean, * *p* ≤ 0.05; NS = non-significant.

**Table 2:** In vitro antioxidant capacity assays in thigh meat of Cobb broiler and Jabalpur color chicken (*n =* 20/group).

Sr No	Antioxidant Capacity Assays	Unit of Measurement	Genetic Group								*p*-Value
			Cobb Broiler				Jabalpur Color Chicken				
			Mean	SE	Minimum	Maximum	Mean	SE	Minimum	Maximum	
1	ABTS (2,2′-azinobis-3- ethylbenzothiazoline-6-sulfonic acid) radical scavenging assay	% Inhibition	29.62	1.27	21.21	37.88	28.48	1.06	21.21	36.36	NS
	TEABTS (Trolox equivalent antioxidative capacity)	Trolox equivalent (TE) µM/g of tissue	3737.50	210.32	2350.00	5100.00	3550.00	175.47	2350.00	4850.00	NS
2	DPPH (1,1-diphenyl-2-picrylhydrazyl) radical scavenging assay	% Inhibition	63.46	0.56	60.17	70.78	67.27	0.63	60.32	71.28	*
3	FRAP (ferric reducing antioxidant power)	mM Fe^2+^/g tissue	19.20	0.31	16.80	21.60	26.83	0.36	24.10	29.80	*
4	Cupric reducing antioxidative capacity (CUPRAC) assay	Trolox equivalent (TE) mM/g of tissue	7.16	0.25	5.08	9.44	7.49	0.30	5.45	10.50	NS
5	ORAC (oxygen radical absorption capacity) assay	Trolox equivalent (TE) µM/g of tissue	762.82	9.19	717.41	795.06	785.95	6.40	751.26	822.93	*
6	MCA (metal chelation activity)	% Inhibition	80.75	0.95	71.25	86.61	63.13	1.87	47.32	81.25	*
		EDTA equivalent activity— EEA µM/g of tissue	4117.96	45.60	3662.98	4398.47	3273.85	89.61	2516.97	4141.91	*
		Carnosine equivalent activity—CEA mM/g of tissue	214.26	2.30	191.27	228.43	171.60	4.53	133.36	215.47	*
7	Superoxide dismutase activity	% Inhibition	89.50	1.34	75.50	99.88	93.69	1.32	77.00	98.50	*

SE: standard error of the mean, * *p* ≤ 0.05; NS = non-significant.

**Table 3:** The concentration of functional biomolecules investigated in the chicken meat.

Sr No	Metabolite	Genotype	Breast		Thigh	
			Mean ± SE	Range	Mean ± SE	Range
1	Carnosine (mg/g of tissue)	Cobb broiler	2.73 ± 0.10	2.11–3.39	0.98 ± 0.03 ^#^	0.78–1.20
		JBC chicken	2.66 ± 0.09	2.15–3.39	1.11 ± 0.04 ^#^	0.74–1.36
2	Anserine (mg/g of tissue)	Cobb broiler	4.85 ± 0.22	3.51–6.33	2.27 ± 0.14 ^#^	1.67–3.14
		JBC chicken	5.11 ± 0.12	4.27–6.10	2.01 ± 0.16 ^#^	1.07–2.92
3	Creatine (mg/g of tissue)	Cobb broiler	3.06 ± 0.12	2.39–3.86	2.56 ± 0.10 ^#^	2.0–3.25
		JBC chicken	2.80 ± 0.12	1.39–2.85	2.46 ± 0.22	1.58–3.40
4	Protein (g/100 g of tissue)	Cobb broiler	21.81 ± 0.10	18.48–24.22	18.31 ± 0.03 ^#^	16.65–20.14
		JBC chicken	25.65 ± 0.39 *	22.06–28.63	19.04 ± 0.23 *^,#^	17.48–20.89

Values are mean ± SE (*n =* 20). * and ^#^ correspond to the significant difference (*p* ≤ 0.05) within a column (Cobb and JBC) and a row (breast and thigh), respectively.

## Data Availability

Data is contained within the article or supplementary material.

## Supplementary Materials

The following supporting information can be downloaded at: https://www.mdpi.com/article/10.3390/foods12132434/s1, Figure S1: Carnosine, anserine, and creatine concentrations of breast and thigh meat; Table S1: Comparative profile of antioxidant capacity of breast and thigh meat among Cobb broiler and Jabalpur color chicken.
