# Supplementary material for: Functional Quality Characteristics of the Meat from a Dual-Purpose Poultry Crossbreed Suitable for Backyard Rearing in Comparison to Commercial Broilers
Source: Foods. 2023 Jun 21;12(13):2434. doi: 10.3390/foods12132434 (PMC10341246; doi:10.3390/foods12132434)
Supplement: Supplementary file 1 [file foods-12-02434-s001.zip › foods-2441196-supplementary.docx]

**Table S1.** Comparative profile of antioxidant capacity of breast and thigh meat among Cobb broiler and Jabalpur color chicken.

Values are mean ± SE (n = 20). Values are mean ± SE (n = 20). *Significant (p ≤ 0.05) difference within a column (Cobb and JBC) and # within a row (Breast and thigh).

1. **ABTS (2,2′-azinobis-3- ethylbenzothiazoline-6-sulfonic acid) radical scavenging assay**

|  | **ABTS** | | **TEABTS** | |
| --- | --- | --- | --- | --- |
|  | (% Inhibition) | | (TE µM/g of tissue) | |
|  | **Breast** | **Thigh** | **Breast** | **Thigh** |
| **Cobb Broiler** | 43.78±1.47 | 29.62±1.27^#^ | 6062.5±257.31 | 3737.5±210.32^#^ |
| **Jabalpur Color** | 52.12±1.36^*^ | 28.48±1.06^#^ | 7375±210.65^*^ | 3550±175.46^#^ |

**2.      DPPH (1,1-diphenyl-2-picrylhydrazyl) radical scavenging assay**

|  | **Breast** | **Thigh** |
| --- | --- | --- |
|  | (% Inhibition) | (% Inhibition) |
| **Cobb Broiler** | 70.56±0.59 | 63.46±0.56^#^ |
| **Jabalpur Color** | 73.92±0.44^*^ | 67.26±0.63^*,#^ |

**3.     FRAP (Ferric reducing antioxidant power)**

|  | **Breast** | **Thigh** |
| --- | --- | --- |
|  | (mM Fe^2+^/g of tissue) | (mM Fe^2+^/g of tissue) |
| **Cobb Broiler** | 15.24±0.40 | 19.20±0.31^#^ |
| **Jabalpur Color** | 22.84±0.25^*^ | 26.82±0.36^*,#^ |

**4.      CUPRAC (Cupric reducing antioxidative capacity) assay**

|  | **Breast** | **Thigh** |
| --- | --- | --- |
|  | (Trolox equivalent (TE ) mM/g of tissue) | (Trolox equivalent (TE ) mM/g of tissue) |
| **Cobb Broiler** | 9.0±0.24 | 7.16±0.25^a#^ |
| **Jabalpur Color** | 12.71±0.32^*^ | 7.49±0.30^#^ |

**5.      ORAC (Oxygen radical absorption capacity) assay**

|  | **Breast** | **Thigh** |
| --- | --- | --- |
|  | (TE µM/g of tissue) | (TE µM/g of tissue) |
| **Cobb Broiler** | 748.56±7.48 | 762.82±9.19 |
| **Jabalpur Color** | 765.82±9.48 | 785.95±6.40^*^ |

**6. Metal Chelation Activity**

|  | **Metal Chelation Activity** | | **EDTA Equivalent activity** | | **Carnosine equivalent activity** | |
| --- | --- | --- | --- | --- | --- | --- |
|  | (% Inhibition) | | (EEA µM/g of tissue) | | (mM/g of tissue) | |
|  | **Breast** | **Thigh** | **Breast** | **Thigh** | **Breast** | **Thigh** |
| **Cobb Broiler** | 53.63±1.79 | 80.75±0.95^#^ | 2819.29±85.84 | 4117.96±45.60^*,#^ | 148.63±4.34 | 214.26±2.30^*,#^ |
| **Jabalpur Color** | 46.3±2.36^*^ | 63.13±1.87^*,#^ | 2468.22±112.81^*^ | 3273.85±89.61^*,#^ | 130.89±5.70^*^ | 171.60±4.53^*,#^ |

**7.      Superoxide dismutase activity**

|  | **Breast** | **Thigh** |
| --- | --- | --- |
|  | (% Inhibition) | (% Inhibition) |
| **Cobb Broiler** | 93.16±1.07 | 89.50±1.34^#^ |
| **Jabalpur Color** | 95.84±0.93 | 93.69±1.32^*^ |

**Figure S1.** Carnosine, anserine, and creatine concentrations of breast and thigh meat. Bars represent mean ± standard error (n = 20). High-performance liquid chromatography (HPLC) linearity range and regression


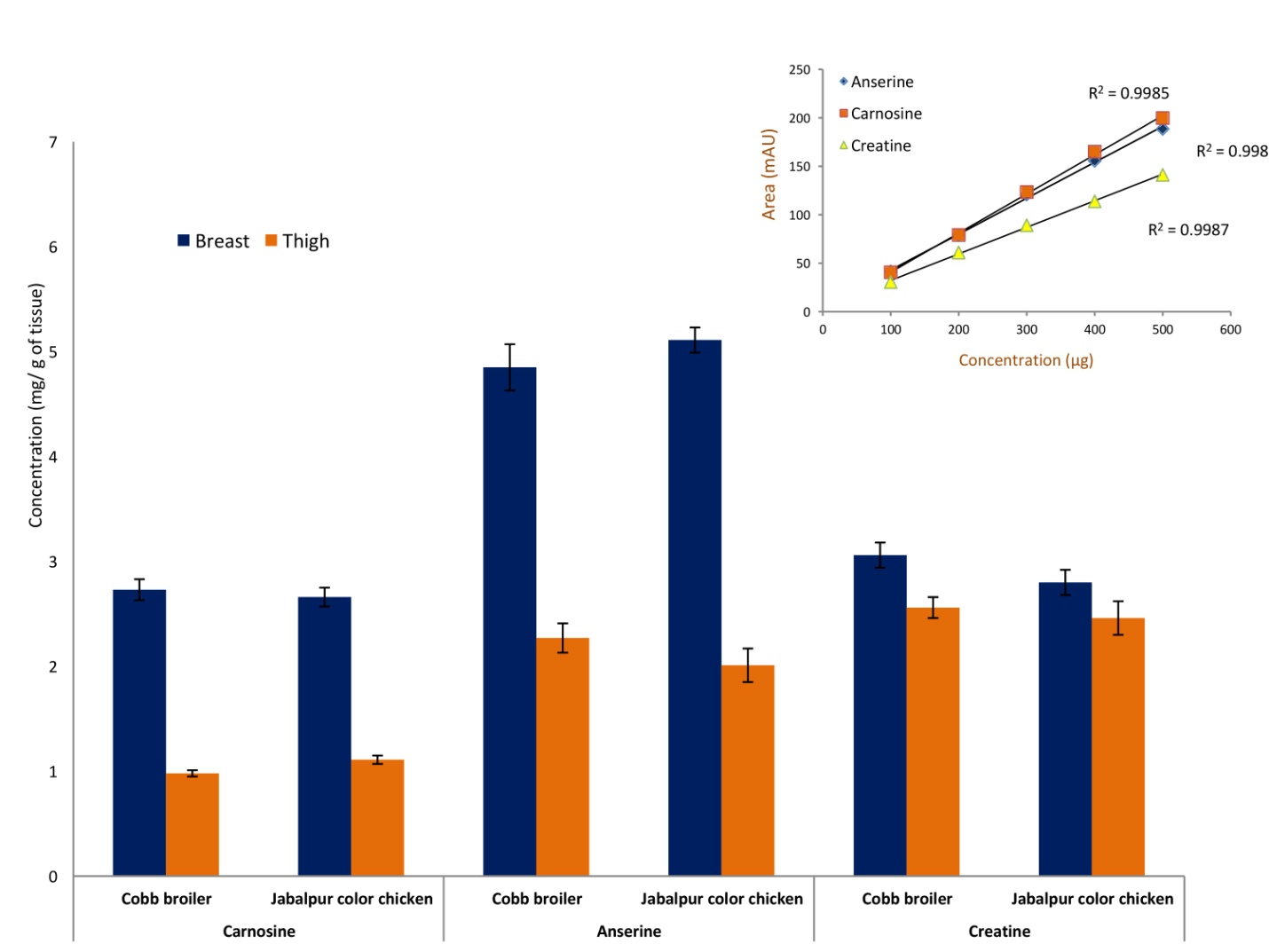


**Standard curves of various Antioxidant capacity assays (*In vitro*)**

| 1 | 2,2′-azinobis-3- ethylbenzothiazoline-6-sulfonic acid (ABTS) assay |
| --- | --- |

| 2 | 1,1-diphenyl-2-picrylhydrazyl (DPPH) assay |
| --- | --- |

| 3 | Ferric reducing antioxidant power (FRAP) assay |
| --- | --- |

| 4 | Cupric reducing antioxidative capacity (CUPRAC) assay |
| --- | --- |

| 5 | Oxygen radical absorption capacity (ORAC) assay |
| --- | --- |

| 6 | Metal chelating activity (MCA) | MCA (%)= (Acontrol – Asample)/Acontrol x 100  A=Iron-ferrozine absorbance at 562 nm |
| --- | --- | --- |
